# Experimental extraction of Young's modulus of gastric tissue with development of spherical, cylindrical, and crowned rollers contact theories

**DOI:** 10.1016/j.heliyon.2024.e31848

**Published:** 2024-05-27

**Authors:** Moein Taheri, Amin Sousanabadi Farahani

**Affiliations:** Department of Mechanical Engineering, Faculty of Engineering, Arak University, Arak, Iran

**Keywords:** AFM, Nanomanipulation, Gastric cancer, Crowned rollers contact models, Young's modulus

## Abstract

Nanotechnology has been considered with the aim of recognizing the structural and mechanical properties as well as improving the treatment and diagnostic process in the field of medicine. The process of nanomanipulation by examining healthy and cancerous tissues in nanoscale is one of the processes used in this field. Therefore, in this article, considering the importance of recognizing the properties of cancerous and healthy tissues in improving the treatment and diagnosis process, one of the most common types of cancer has been studied. Young modulus has been used as a parameter in the diagnosis of cancerous tissue and its value has been calculated for gastric cancerous tissue. To achieve this goal, atomic force microscopy (AFM) was used during the manipulation process. This tool with the ability to study cancerous tissues in different environments and with the least amount of damage to the target tissue, is one of the effective tools in the field of nanomanipulation. The parameter studied in this study is the geometry of gastric cancer tissue. Therefore, the simulations have been performed by considering contact models with spherical, cylindrical and crowned rollers geometries. The force-indentation depth diagram for gastric tissue is plotted experimentally and compared with theoretical results. According to the experimental work done after reviewing the recorded topographic images, the approximate range of the Young's modulus value for gastric tissue has been calculated according to different geometries. Since the geometry of the crowned rollers is closer to the geometry of the gastric tissue, it has a higher accuracy and the values of the Young's modulus have been calculated according to this geometry in the range of 316–310 KPa.

## Graphical abstract

**Figure:**
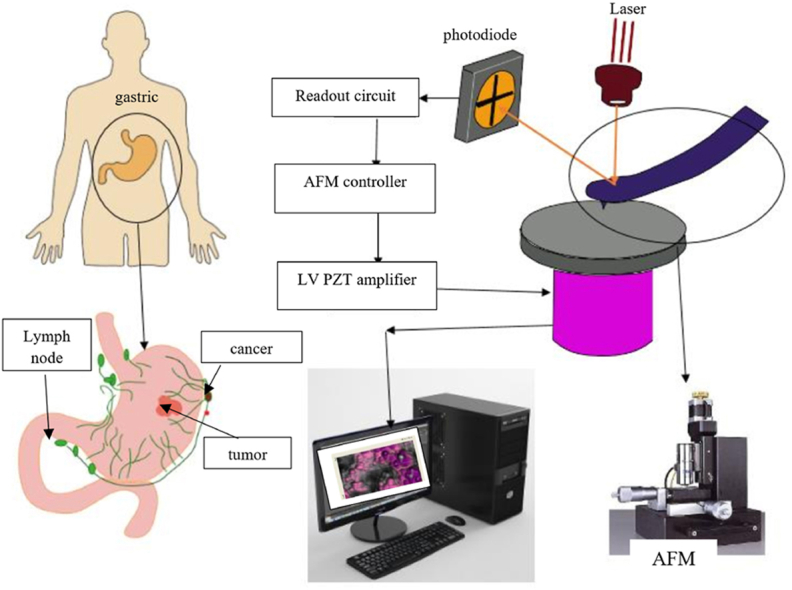

## 1 Introduction

The study of biological tissues, the analysis of the mechanical properties of particles at the nanoscale, and the study of the electrical properties of materials are among the applications of atomic force microscopy in various industries and sciences. Therefore, extensive studies have been conducted on the advantages, disadvantages, and performance improvements of this tool. Nanomanipulation is a field that uses AFM. Reasons for using this tool, especially in the study of biological tissues such as cancerous tissues, include high-resolution imaging and no environmental restrictions. Using this tool, various textures have been studied and mechanical properties such as strength, Young's modulus, and electrical properties have been investigated. Note that the general process of research in the field of manipulation is examined in two phases, and all environmental and geometric factors are examined so that the results are closer to reality and provide better results (see Fig. 3).

Tschaikowsky et al. used a combination of AFM and fluorescence microscopy to describe biological tissue. They selected a millimeter-sized tissue sample by micrometer-scale fluorescence microscopy and then mapped it to nanometer accuracy under AFM under natural conditions. The tissue anLATalyzed was cartilage [1]. Verma et al. used an atomic force microscope to investigate the mechanical properties and surface charge properties of chitosan/alginate-based films for biomedical applications. In this experiment, by changing the concentration of chitosan and alginate, films with different surface charge densities and mechanical properties were created, and the surface charge density of these films was determined using an analytical model on the force curves obtained from atomic force microscopy (AFM) [2]. Li et al. Studied the dynamic properties of the nanowire surface using an atomic force microscope. The motion of the microscope cantilever is modeled as a spring according to the Hertz contact model, then the system resonance behaviors are studied theoretically and experimentally and the theoretical resonance frequencies are well matched with the experimental frequencies. Dynamic studies based on the proposed model show that the specific surface properties of nanoscale nanowires are mainly due to the distributed force due to the residual surface stress [3]. Cheong et al. Used AFM to study electrical properties such as charge diffusion, dielectrics, surface potentials, conductivity, and piezoelectric in biomolecules, biological membranes, cells, tissues, and other biological samples. They have also analyzed the theories and achievements of different AFM modes in this field [4]. Knotek et al. Evaluate various applications of atomic force microscopy (AFM) such as phase shift image, atomic force acoustic microscope (AFAM) and force measurement to model low molecular weight biomimetic groups on the polymer. Peptide molecules are localized by detecting gold nanospheres bound by AFM. AFM methods have been able to monitor local mechanical properties and have been the most efficient methods for detecting gold nanomarkers [5].

Because of the limitations of standard AFM probes for studying the mechanical properties of large cells or three-dimensional multicellular masses, Andolfi et al. Designed and built flat AFM macro probes compatible with commercial AFM tools. The probes consist of a large flat compression plate attached to the chip by two flexible arms, the mechanical properties of which can be adjusted for specific biological applications. To validate this experimental structure and measure its viscoelasticity, large spherical biological systems with a diameter of more than 100 μm have been designed and the results have been studied [6]. Luo et al. Measured cell stiffness using afm. The most common method of measuring stiffness has been to calculate the Young cell modulus, which changes with the incidence of cancer and has been considered as a biomarker of cell motility, especially in estimating cancer cell metastasis. They also discuss the main determinants of AFM-determined cell stiffness with a focus on the cytoskeleton, nuclear stiffness, and methodological [7]. By integrating carbon nanotubes with an atomic force microscope, TermehYousefi et al. developed high-performance nanoscopes for biological single-cell analysis. Strong mechanical properties, nanoscale diameters, and their ability to be functionalized by chemical and biological components are among the reasons for selecting carbon nanotubes [8]. Stylianou et al. used an atomic force microscope (AFM) to examine the thin layers of collagen characterized by ultraviolet (UV) light. These films have also been used as laboratory culture media to study UV-induced changes in fibroblasts. Finally, the results show that for short irradiation times, spectroscopic studies (fluorescence/absorption), optical degradation occurred, and AFM imaging has shown changes in surface roughness [9].

Saijo et al. Used a scanning microscope to examine the cancerous gastric tissue Experimental work has been done by them on five different types of gastric cancer, and the results have shown the effect of intercellular binding and intracellular chemical components on ultrasonic properties [10]. Bu et al. Measured atomic force microscopy, creep deformation, and stress relaxation of six human cell lines. Comparing the results of experimental and theoretical experiments, they found that normal human liver cell lines (L02), liver cancer (HepG2), liver star (LX2), and gastric cancer (NCI–N87) are linear viscoelastic substances, whereas human (GES-1) and gastric cancer (SGC7901) cell lines are nonlinear [11]. Codan et al. used AFM to investigate the relation between the Young's modulus of living and healthy cells and to study the mechanical properties of two cell lines, mouse embryonic fibroblasts (NIH/3T3) and human epithelial cancer cells (SW-13). Both living cells and cells stabilized with paraformaldehyde were examined. This analysis quantifies the difference between the Young's modulus for these two conditions and provides a coefficient for their correlation [12]. Taheri and Batahi studied the mechanical study of gastric cancer tissue by modeling and simulating the manipulation process. Their experimental work was performed under an atomic force microscope, and the simulations were performed using contact mechanics and Hertz and GKR contact models. By drawing experimental and simulated graphs and comparing them, 325 kPa was calculated for the Young modulus of gastric cancer cells [13]. Bo et al. reported the use of atomic force microscopy in biological studies. The reasons for this application have been high-resolution imaging and manipulation of living cells in a liquid medium. In addition, to cover AFM deficiencies, the results of AFM research along with other technologies are introduced, and the development trend of AFM in cell biology research is predicted [14]. Zhang et al. calculated the cell membrane capacity of gastric cancer. One of the reasons for examining this cellular property is the change in this property during cancer and the possibility of detecting cancerous tissue [15]. Vaiani et al. proposed an alternative approach for extracting common mechanical parameters, such as Young's modulus, from cellular components, beginning with AFM nanodimensional measurements performed on human mesenchymal stem cells. Geometric modeling was performed in a virtual environment, and the force-depression curve is plotted. Finally, the results show that this algorithm can detect the material properties of various intracellular components such as the cortex and cytoskeleton. The numerical results predicted by the elastic lattice model are then compared with the results obtained from the Hertz contact theory and finite element method (FEM) for similar case studies, which show optimal agreement and greatly reduced computational cost [16].

Ridzuan Ahmad et al. studied the nanomanipulation of yeast cells to determine their mechanical properties. Analysis has shown that cell size can affect its mechanical strength. As the cell size increases, the pressure force required to penetrate its wall also increases. Furthermore, from these experimental results, it can be concluded that the force required to penetrate a cell does not depend only on the internal factor, cell size, but also on external factors, such as environmental conditions [17]. Korayem et al. analyzed the contact mechanics of four different materials to investigate the depression depth parameter during manipulation. Contact models used for spherical and elliptical geometry. Comparing spherical and elliptical geometries, the depth of depression for spherical geometry is greater than that for elliptical geometry because of the presence of eccentricity in elliptic contact models, which do not exist in spherical geometry. Based on existing experimental work, the jang-wang model is the most suitable model for application at the particle-bed contact point [18]. Shen and colleagues have developed a method for measuring single-cell stiffness based on nano-needles and nano-manipulation. They prepared wild-type yeast cells (W303) and placed them on the sample stage inside a peripheral scanning electron microscope (ESEM) chamber. Single-cell stiffness is determined based on this deformation information. Finally, the results show that the stiffness of a cell decreases with increasing humidity [19]. Korayem et al. Used an atomic force microscope to study the force applied to deform a DNA cell during the manipulation process. Modeling of contact mechanics was performed using the GCR contact model. Finally, by comparing the results with gold nanoparticles, they conclude that the applied force for deformation in the DNA molecule was less than that of gold nanoparticles [20]. Hou et al. Reviewed recent advances in nanomanipulation and its applications in medicine. These improvements include mechanical properties, structural transfer, position/state regulation, intramolecular/intermolecular interactions of biological objects [21]. Korayem and Khaksar have studied the spherical and elliptical geometries of nanoparticles during manipulation. Dynamic modeling and simulation have been performed using Hertz, JKR and Jamari theories. Also, considering the approximate relation of the Hunter impact, the effects of the impact on motion are also considered. Finally, the results are calculated after 3 s under constant, linear, second-order and Sin forces for elliptical and cubic nanoparticles [22].

As mentioned, atomic force microscopy has been used to extract mechanical properties such as modulus, mechanical strength, cell membrane capacity, and more in biological tissues such as cancer. This article specifically addresses the Young's modulus and uses it as a biomarker in the diagnosis of cancer. In previous research, parameters such as the contact model have been extensively studied, but it is important to determine the true geometry of the cancerous tissue before studying other parameters because it directly affects the contact [23].

Therefore, in this paper, three spherical geometries, cylindrical and chamfered cylinders have been studied and the Hertz and JKR contact equations have been developed according to these geometries. All three geometries are shown schematically in the recorded images of the gastric cancer tissue. The experimental results are plotted and compared with theoretical graphs, and the values of the Young ‘s modulus are calculated.

## 2 Modeling

**Fig. 1:**
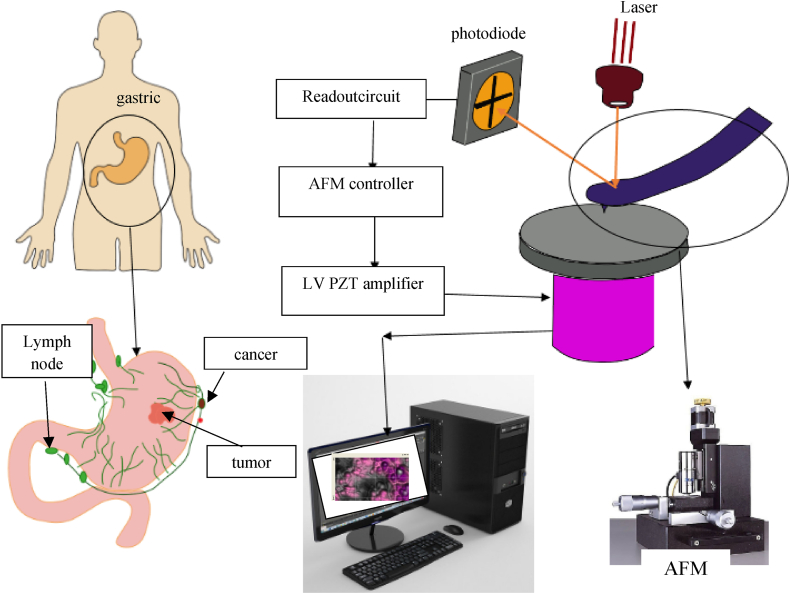
The process of the manipulation process.

**Fig. 2:**
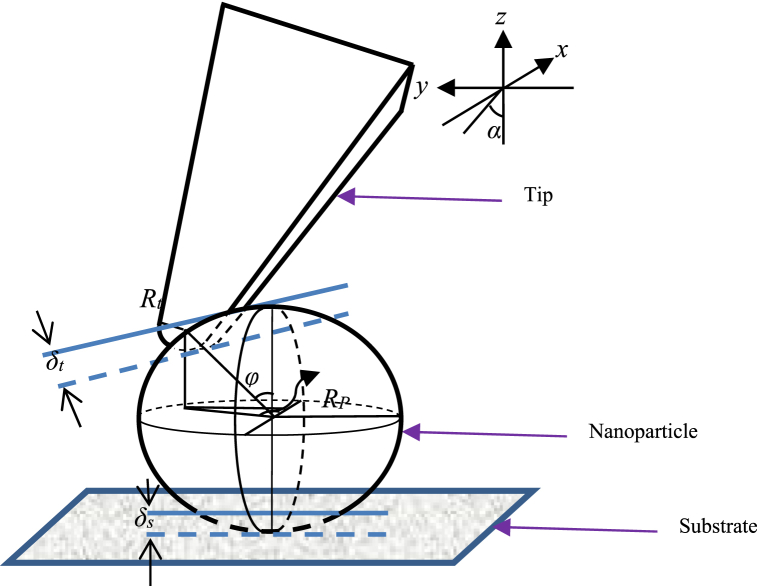
Schematic of contacts between particles, tip and substrate [24].

In this paper, with the aim of extracting the Young modulus of gastric cancer tissue, the manipulation process is modeled and simulated. Extraction of this mechanical property is performed in the second phase of the manipulation. For this purpose, after extracting the force and critical time in the first phase, the movement begins and the process enters the second phase. The probe tip of the atomic force microscope is in contact with the particle, and the increase in force on the cantilever causes a change in the displacement of the nanoparticle. The information recorded in this step is processed by a computer, and force–depth diagrams of the nanoparticles are drawn. It should be noted that, as shown in Fig. 1, the information was extracted by laser light irradiation on an atomic force microscope and its reflection in a photodiode. Because of the focus of this paper on contact mechanics models, it is necessary to identify the contact areas in nanoparticle manipulation. Therefore, the contact is examined in two areas, first in the contact between the probe tip and the particle and the other in the contact area of the particle and the substrate (see Fig. 2).

**Fig. 3:**
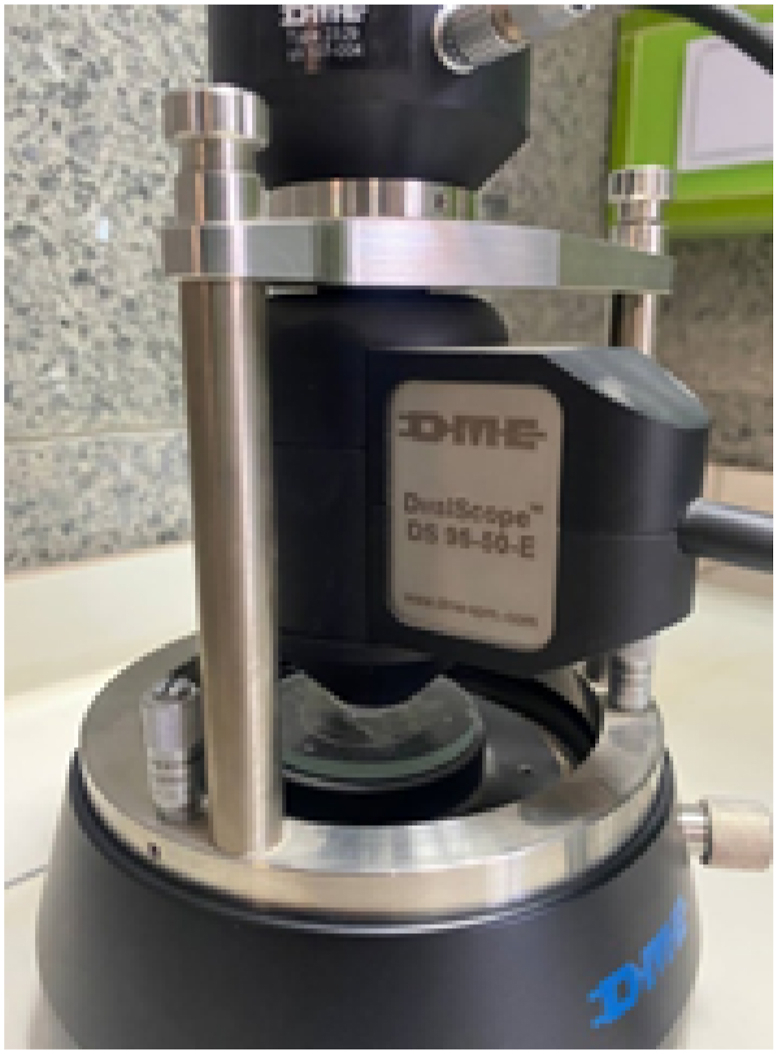
Atomic microscope for dualscope company.

### 2.1 Contact models

In this research, the mechanics of nanoparticle contact have been investigated in three parts: spherical, cylindrical and crowned roller. The Hertz and JKR contact models used were analyzed at two levels of particle–tip and particle-substrate contact.

#### 2.1.1 Hertz contact model

The Hertz contact model was one of the first theories to be studied in contact mechanics and was established assuming the absence of adhesion force. General relation for the force and radius of contact as well as the depth of penetration are given in relations 1 to 3. These equations are established assuming that the geometry of the nanoparticle is spherical [25].

$$\begin{array}{ccc} \text{Spherical}\;\text{geometry} & \begin{array}{c} F_{\left(adh\right)Hertz}=0\\ \;a_{Hertz}={\left(\frac{\bar{R}F}{K}\right)}^{1/3}\\ {\;\delta }_{Hertz}=\frac{a_{Hertz}^{2}}{\bar{R}} \end{array} & \begin{array}{c} \left(1\right)\\ \left(2\right)\\ \left(3\right) \end{array} \end{array}$$

New contact equations have been obtained by developing equations in the biological environment and by separating contact surfaces into nanoscale dimensions. At the contact of a particle and a tip of AFM is considered equal to $F=P_{Z}\;\cos\;\varphi +P_{Y}\;\sin\;\varphi$ and the contact radius is equal to $\bar{R}=\frac{R_{p}\times R_{t}}{R_{p}+R_{t}}$, and the equations are rewritten as equations 4 to 6.

$$\begin{array}{ccc} \text{Tip}-\text{particle} & \begin{array}{c} F_{\left(adh\right)Hertz}=0\\ a_{Hertz}={\left(\frac{\left(R_{p}\times R_{t}\right)\left[P_{Z}\;\cos\;\varphi +P_{Y}\;\sin\;\varphi \right]}{\left(R_{p}+R_{t}\right)K}\right)}^{1/3}\\ \delta_{Hertz}=\frac{a_{Hertz}^{2}\left(R_{p}+R_{t}\right)}{R_{p}\times R_{t}} \end{array} & \begin{array}{c} \left(4\right)\\ \left(5\right)\\ \left(6\right) \end{array} \end{array}$$

The equivalent force and radius at the particle-plane contact are assumed to be equal to $F=P_{Z}$ and $\bar{R}=R_{p}$, and the Equations are rewritten as Equations 7 to 9.

$$\begin{array}{ccc} \text{Substrate}-\text{particle} & \begin{array}{c} F_{\left(adh\right)Hertz}=0\\ a_{Hertz}=\frac{4\;\mathrm{PR}}{\pi E^{*}}\\ \delta_{Hertz}=\frac{a_{Hertz}^{2}}{R_{p}} \end{array} & \begin{array}{c} \left(7\right)\\ \left(8\right)\\ \left(9\right) \end{array} \end{array}$$

Considering the cylindrical geometry for gastric cancer tissue and defining the parameters $\delta_{Hertz}$ penetration depth, a, contact radius, R particle radius, P external applied force, L * cylindrical length, $E^{*}$ modulus of effective elasticity, $\frac{1}{E^{*}}=\frac{m_{t}}{2}\left(\frac{1-\nu_{1}^{2}}{E_{1}}+\frac{1-\nu_{2}^{2}}{E_{2}}\right)$ and $E_{1}$, $E_{2}$ modulus of Young and $\nu_{1}$, $\nu_{2}$ Poisson coefficients at the two Poisson coefficients, *mt* is a constant and dependent parameter on the geometry of the tip (mt = 1 for a cylindrical geometry, *mt* = 1.5 for spherical geometry and *mt* = 2 for conical forms) [26].

contact equations are calculated as 10 to 12 Equations. After applying the conditions of the biological environment and placing the equivalent force and radius according to the contact surface, the Equations will be rewritten as Equations 13 to 18 [27].

$$\begin{array}{ccc} \text{Cylindrical}\;\text{geometry} & \begin{array}{c} F_{\left(adh\right)Hertz}=0\\ a_{Hertz}={\left(\frac{\bar{R}F}{K}\right)}^{1/3}\\ \delta_{Hertz}=\frac{a^{2}}{2R}\left\{2\;\ln\left(4R/a\right)-1\right\} \end{array} & \begin{array}{c} \left(10\right)\\ \left(11\right)\\ \left(12\right) \end{array} \end{array}$$

$$\begin{array}{ccc} \text{Tip}-\text{particle} & \begin{array}{c} F_{\left(adh\right)Hertz}=0\\ a_{ertz}={\left(\frac{\left(R_{p}\times R_{t}\right)\left[P_{Z}\;\cos\;\varphi +P_{Y}\;\sin\;\varphi \right]}{\left(R_{p}+R_{t}\right)K}\right)}^{1/3}\\ \delta_{Hertz}=\frac{a^{2}\left(R_{p}+R_{t}\right)}{2\left(R_{P}\times R_{t}\right)}\left\{2\;\ln\left(4\left(\frac{R_{p}\times R_{t}}{R_{p}+R_{T}}\right)/a\right)-1\right\} \end{array} & \begin{array}{c} \left(13\right)\\ \left(14\right)\\ \left(15\right) \end{array} \end{array}$$

$$\begin{array}{ccc} \text{Substrate}-\text{particle} & \begin{array}{c} F_{\left(adh\right)Hertz}=0\\ a_{Hertz}=\frac{4\;\mathrm{PR}}{\pi E^{*}}\\ \delta_{Hertz}=\frac{a^{2}R_{p}}{2}\left\{2\;\ln\left(4R_{p}/a\right)-1\right\} \end{array} & \begin{array}{c} \left(16\right)\\ \left(17\right)\\ \left(18\right) \end{array} \end{array}$$

#### 2.1.2 JKR contact model

The JKR contact theory has been studied in general and in Equations 19 to 21. These equations are established by considering the spherical geometry [28].

SphericalgeometryF(adh)JKR=(6πωKaJKR3)1/2aJKR=[R‾K[F+3πR‾ω+(6πR‾ωF+(3πR‾ω)2)1/2]]13δJKR=aJKR2R‾−8πωaJKR3K(19)(20)(21)

Considering the equivalent radius and the applied force in the biological environment for the cancerous tissue studied, the JKR contact equations were rewritten as 22 to 27 and were divided into two levels: particle–tip and particle-substrate.

Tip−particleF(adh)JKR=(6πω*KaJKR3)1/2aJKR=[(Rp×Rt)(Rp+Rt)K((PZcosφ+PYsinφ+3π(Rp×Rt)Rp+Rtω*+[6π(Rp×Rt)Rp+Rtω*(PZcosφ+PYsinφ)+(3π(Rp×Rt)Rp+Rtω*)2]1/2)]13δJKR=aJKR2(Rp+Rt)Rp×Rt−8πω*aJKR3K(22)(23)(24)

Substrate−particleF(adh)JKR=(6πω*KaJKR3)1/2aJKR=[RpK[PZ+3πRpω*+[6πRpω*PZ+(3πRpω*)2]1/2]13δJKR=aJKR2RP−8πω*aJKR3K(25)(26)(27)

The relations between the force and the radius of contact and the depth of penetration are written in cylindrical geometry and the JKR contact model as 28 to 30 Equation [29].

$$\begin{array}{ccc} \text{Cylindrical}\;\text{geometry} & \begin{array}{c} F_{\left(adh\right)JKR}=\left(\frac{\pi E^{*}a^{2}}{4R}-\sqrt{2\pi E^{*}\omega }\right)\\ a_{JKR}={\left(\frac{2R^{2}\omega }{\pi E^{*}}\right)}^{\frac{1}{3}}\\ \delta_{JKR}=\frac{a_{JKR}^{2}}{\bar{R}}-\sqrt{\frac{8\pi \omega a_{JKR}}{3K}} \end{array} & \begin{array}{c} \left(28\right)\\ \left(29\right)\\ \left(30\right) \end{array} \end{array}$$

For the contact between the particle and the tip, assuming the applied force $F=F_{Z}\;\cos\;\varphi +F_{Y}\;\sin\;\varphi$ and the coefficient of adhesion between the tip and the particle in the biological fluid environment $\omega =\omega_{tLp}=\omega_{tp}+\omega_{LL}-\omega_{tL}-\omega_{pL}$, Equations 31 to 33 are obtained.

$$\begin{array}{ccc} \text{Tip}-\text{particle} & \begin{array}{c} F_{\left(adh\right)JKR}=\left(\frac{\pi E^{*}a_{JKR}^{2}}{4R}-\sqrt{2\pi E^{*}\left(\omega_{tp}+\omega_{LL}-\omega_{tL}-\omega_{pL}\right)}\right)\\ a_{JKR}={\left(\frac{2R^{2}\left(\omega_{tp}+\omega_{LL}-\omega_{tL}-\omega_{pL}\right)}{\pi E^{*}}\right)}^{\frac{1}{3}}\\ \delta_{JKR}=\frac{a_{JKR}^{2}}{R}-\sqrt{\frac{8\pi \left(\omega_{tp}+\omega_{LL}-\omega_{tL}-\omega_{pL}\right)a_{JKR}}{3K}} \end{array} & \begin{array}{c} \left(31\right)\\ \left(32\right)\\ \left(33\right) \end{array} \end{array}$$

In the contact of the particle-substrate, the force of action is considered equal to $F=F_{Z}$ and the contact equations 34 to 36 are obtained.

$$\begin{array}{ccc} \text{Substrate}-\text{particle} & \begin{array}{c} F_{\left(adh\right)JKR}=\left(\frac{\pi E^{*}a^{2}}{4R_{p}}-\sqrt{2\pi E^{*}\omega }\right)\\ {\;a}_{JKR}={\left(\frac{2{R_{p}}^{2}\omega }{\pi E^{*}}\right)}^{\frac{1}{3}}\\ \delta_{JKR}=\frac{a_{JKR}^{2}}{R_{p}}-\sqrt{\frac{8\pi \omega a_{JKR}}{3K}} \end{array} & \begin{array}{c} \left(34\right)\\ \left(35\right)\\ \left(36\right) \end{array} \end{array}$$

#### 2.1.3 Crowned roller contact model

In contact equations, assuming the geometry of a Crowned roller, the geometry of these particles is considered as the sum of spherical and cylindrical geometry, and the penetration depth equation will be calculated as Equation (37) [30].

(37) $$\delta_{Hertz}=\frac{P-2\left(\pi \kappa E^{\prime }{\left[\frac{2\varepsilon R}{9}{\left(\frac{\delta_{c}}{\zeta }\right)}^{3}\right]}^{\frac{1}{2}}\right)}{2C\pi E^{\prime }}\left\{\ln\frac{8\text{CR}\pi E^{\prime }}{P-2\left(\pi \kappa E^{\prime }{\left[\frac{2\varepsilon R}{9}{\left(\frac{\delta_{c}}{\zeta }\right)}^{3}\right]}^{\frac{1}{2}}\right)}-1\right\}$$

## 3 Experimental design

This section examines the experimental work process and introduces the results of the contact models used in the simulation.

### 3.1 Simulation assumptions

The AFM geometric constants and mechanical properties are shown in Table 1. These are the standard values which are usually used for simulating the AFM work process. L, t and W are Length, thickness and width of the cantilever and H is the probe height. Also $R_{t}$ is the tip radius. E and G are Young and Shear modulus and ν is Poisson's coefficient.

**Table 1:** AFM geometric constants and mechanical properties.

L(μm)	w(μm)	t(μm)	H(μm)	$R_{t}\left(nm\right)$	E(GPa)	G(GPa)	ν
225	48	1	12	20	169	66.54	0.27

### 3.2 Experimental method

At this stage, gastric cancer tissue is prepared for examination. The washing of these tissues was done after separation and after placing the stabilizer for 60 s, the desired tissue was washed with salt in three stages and finally, the tissue was dried. The prepared tissues were stored in an antibacterial solution with 4 % formalin at a temperature below 10 °C for 2 days and then transferred to the laboratory.

### 3.3 Atomic force microscope data

**Fig. 4:**
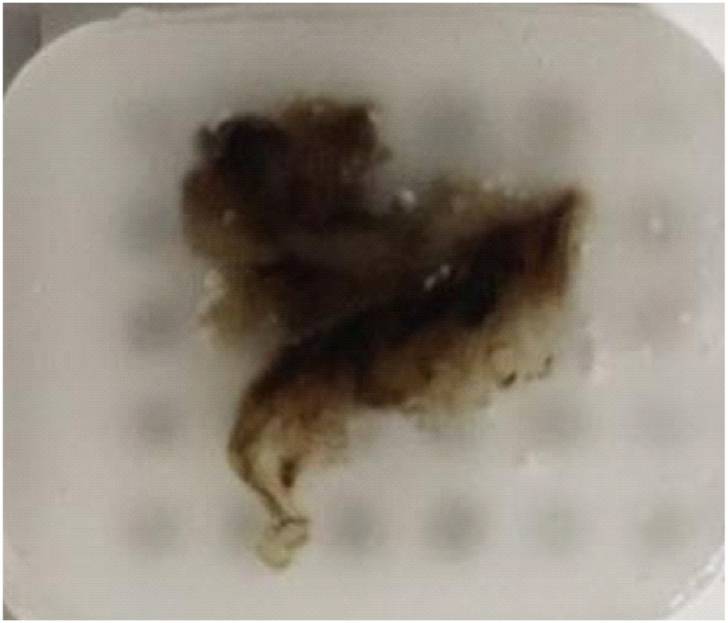
Tissue samples used experiments.

**Fig. 5:**
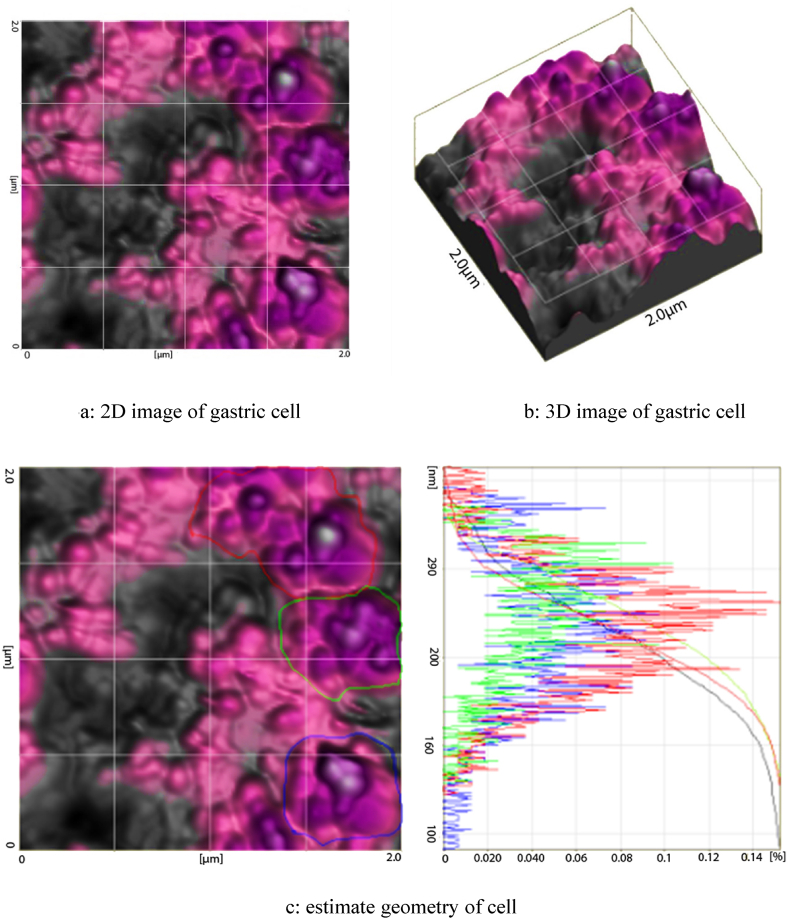
Topographic images of cell.

One of the applications of atomic force microscopy is the extraction of the properties of cell tissues (see Fig. 4). Therefore, the first step in collecting information is to explore the desired texture surface and take topographic images. In this study, 2D and 3D images of the gastric cancer tissue are shown in Fig. 5-a and 5-b. Another goal of this study was to study different geometries for gastric cancer. Therefore, Fig. 5-C shows different ranges for averaging cell height and determining different geometries in different parts of the cancer tissue.

**Fig. 6:**
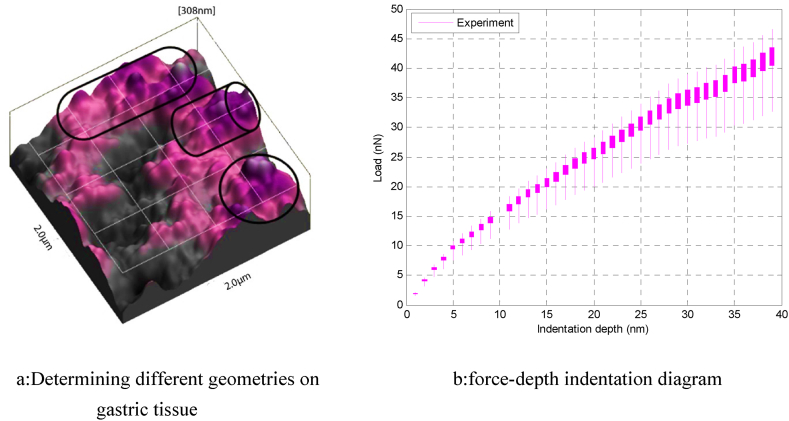
Experimental results of gastric tissue nanomanipulation.

The first step in simulating gastric cancer tissue manipulation and calculating Young's modulus is to consider different geometries for use in contact models. Therefore, in Fig. 6-a of the topographic images recorded by the atomic force microscope, spherical, cylindrical and Crowned rollers geometries are considered. Another result of this study is the drawing of a force–depth penetration diagram for gastric cancer tissue experimentally. Therefore, finally, the diagram of Fig. 6-b is compared with the simulation results and the appropriate modulus is calculated.

### 3.4 Estimation of Young's modulus with different contact models

**Fig. 7:**
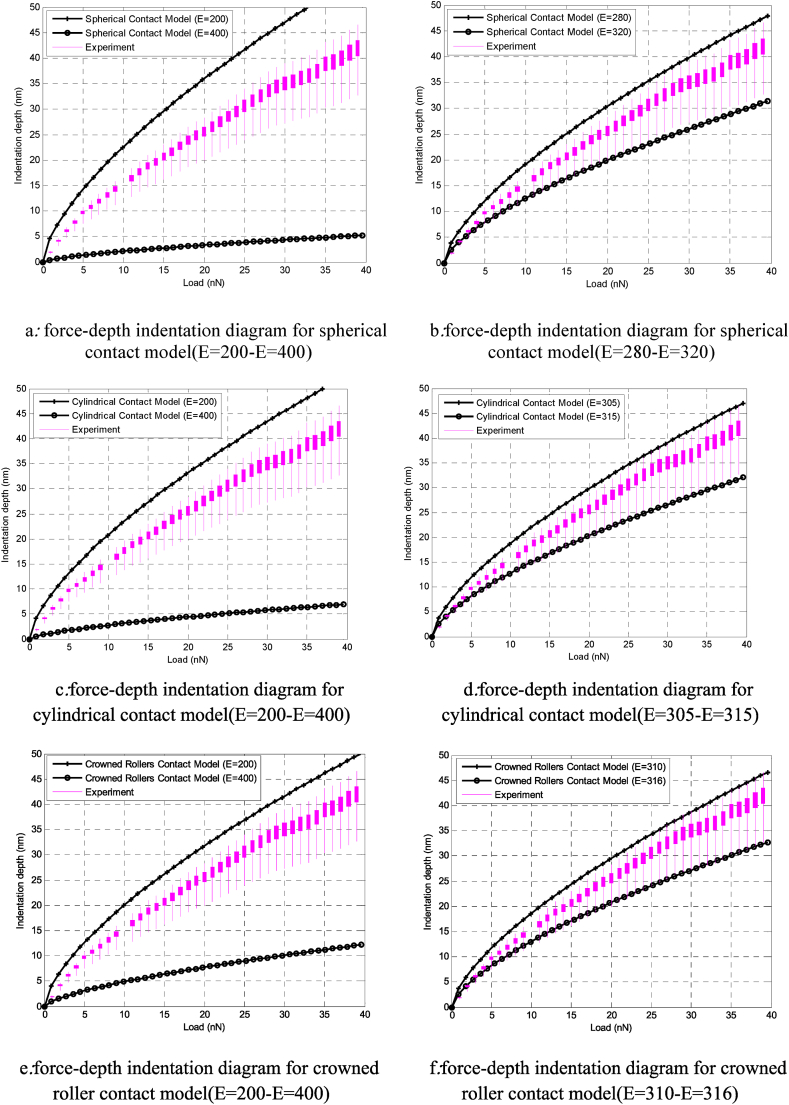
Young's modulus of gastric cancer cells using different contact model.

As mentioned in Fig. 6-b, the first step in extracting the Young ‘s modulus of cellular tissue is to plot the experimental force-depth diagram. In this study, the most important parameter is cell geometry. Therefore, different spherical, cylindrical and Crowned rollers geometries have been considered in contact models and in simulation of manipulation process. The next step is to determine the approximate range of the Young ‘s modulus for each geometry. In this research, initially the range of 200–400 KPa is considered for all geometries. Then, with decreasing amplitude, the approximate values of the Young ‘s modulus of gastric cancer tissue are considered. Considering the spherical geometry, in Fig. 7-b, the range 280–320 KPa for the Young's modulus of gastric tissue is calculated. The matching of the experimental and theoretical diagrams for the cylindrical geometry occurred in the range of 315–305 (Fig. 7-d). Considering the geometry of the crowned rollers for gastric cancer tissue in Fig. 7-f, the theoretical and experimental diagrams are more in the range of 316–310 KPa.

## 4 Validation

**Fig. 8:**
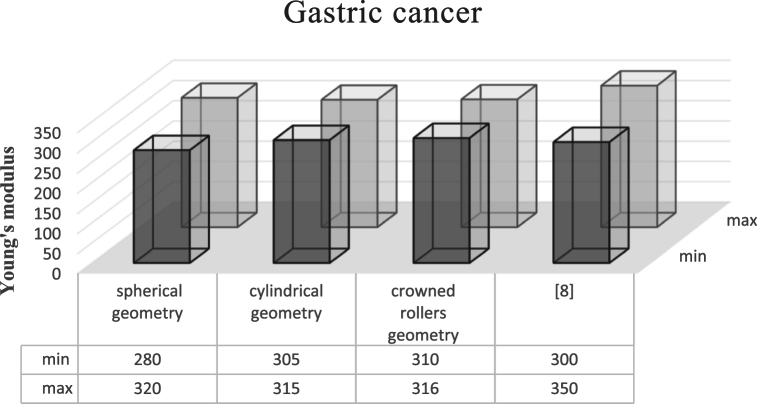
Validation of gastric cancer cells.

To validate the values calculated in this paper, the diagrams in Fig. 8 are drawn. As can be seen, the results of this study have been compared with three spherical, cylindrical, and crowed roller geometries with the values calculated in reference [11]. In Ref. [11], the contact theory is considered as Hertz and JICR, and the cell geometry is assumed to be spherical. The difference between these values and the spherical geometry of this research can be explained by different environmental conditions and tools. It should be noted that the crowed roller geometry gives better results due to being closer to the real geometry.

## 5 Conclusions

The study of the properties and structure of materials in various fields of industry and medicine is one of the innovative researches in the field of nanotechnology. In addition, due to the importance of human health, the study of diseases such as cancer has been considered by many researchers in this field. The Young modulus parameter changes with the conversion of a healthy cell to a cancer cell and is easily measurable. Therefore, in this study, the value of this parameter was calculated for gastric cancer tissue. One of the effective parameters in calculating Young's modulus of cancerous and healthy tissues is the study of different geometries in the mechanics of cell view. Therefore, in this article, according to the experimental work performed and the images recorded by the atomic force microscope, three spherical, cylindrical and crowned rollers for gastric cancerous tissue are considered. Then, the experimental results obtained from the recording of displacement and force changes are plotted as an empirical graph of the forcedepth of indentation. In the simulation, after applying the approximate range of Young's modulus for all three geometries, the Young's modulus of gastric cancer tissue is calculated more accurately. Finally, due to the closeness of the geometry of the studied cancerous tissue in the form of a crowned rollers, the values of this simulation are considered as a reference for the value of the Young's modulus of gastric cell tissue. This value is in the range of 316–310 KPa. It is suggested that in future research and considering the importance of recognizing the mechanical properties of cancerous and healthy tissues, other factors of the manipulation process, such as resistive forces such as adhesion, friction, geometric parameters of the atomic microscopy beam, other contact models and other factors affecting the research process, should be examined.

## CRediT authorship contribution statement

**Moein Taheri:** Writing – review & editing, Project administration, Methodology, Conceptualization. **Amin Sousanabadi Farahani:** Writing – review & editing, Writing – original draft.

## Declaration of competing interest

The authors declare that they have no known competing financial interests or personal relationships that could have appeared to influence the work reported in this paper.

## List of symbols

^*a*^Hertz: Contact radius in Hertz model
^*a*^_*JKR*_: Contact radius in JKR model
^*F*^_*(adh)Hertz*_: Adhesion force in Hertz model
^*F*^_*(adh)JKR*_: Adhesion force in JKR model
K: Effective modulus of elasticity
P: Externally applied force
R: Particle's equivalent radius
*δ*_*Hertz*_: *Indentation depth in Hertz model*
*δ*_*JKR*_: *Indentation depth in JKR model*
*φ*: Probe-particle contact angle
$\bar{R}$: *Effective radius of two contacted surfaces*
R_1_,R_2_: radius of the two surfaces in contact
F: Normal force in constant area
ν_1_, ν_2_: Poisson's ratio of probe
mt: mt is a constant and dependent parameter on the geometry of the tip
ω: work of adhesion
ζ: Complete elliptic integral of first kind
R_P_: Micro/nanoparticle radius
R_T_: *Spherical tip radius*
P_y_: *External force along the vertical axis*
P_z_: *External force along the horizontal axis*
G: *modulus of rigidity*
H: Height of the probe
L: *Length of probe*
t: *Thickness of probe*
w: *Width of probe*
